# Description of *Tabanusrondoniensis* (Diptera: Tabanidae), a new species of horsefly from the State of Rondônia, Brazil

**DOI:** 10.3897/BDJ.10.e76904

**Published:** 2022-04-26

**Authors:** Augusto Loureiro Henriques, Tiago Kütter Krolow, Tallita Beatriz de Oliveira Zamarchi, Luís Marcelo Aranha Camargo

**Affiliations:** 1 Instituto Nacional de Pesquisas da Amazônia, Manaus, Brazil Instituto Nacional de Pesquisas da Amazônia Manaus Brazil; 2 Universidade Federal do Tocantins, Porto Nacional, Brazil Universidade Federal do Tocantins Porto Nacional Brazil; 3 Fundação Oswaldo Cruz, Instituto Leônidas e Maria Deane, Manaus, AM, Brasil., Manaus, Brazil Fundação Oswaldo Cruz, Instituto Leônidas e Maria Deane, Manaus, AM, Brasil. Manaus Brazil; 4 Universidade de São Paulo, Instituto de Ciências Biomédicas 5, Monte Negro, RO, Brasil., Monte Negro, Brazil Universidade de São Paulo, Instituto de Ciências Biomédicas 5, Monte Negro, RO, Brasil. Monte Negro Brazil

**Keywords:** Tabanini, horseflies, Neotropics, Amazon Region, new taxon

## Abstract

**Background:**

The genus *Tabanus* Linnaeus has a worldwide distribution and is the richest in species; however, it is probably not monophyletic. In the Neotropical Region, its richness is certainly underestimated, mainly due to the large number of species and the absence of recent taxonomic revisions.

**New information:**

We describe *Tabanusrondoniensis* sp. n. from the State of Rondônia, Brazil, based on a conspicuous tabanid species possibly related to the *T.nebulosus* species group. Diagnosis, discussion and illustrations are also provided.

## Introduction

Tabanids are flies of medical and veterinary importance because of the haematophagous habit and the potential transmission of pathogens (Baldacchino et al. 2014). It is a large group, with some 4,500 species occupying the most varied regions of the planet. The genus *Tabanus* is the richest group of species, with about 1,350 described species (Pape and Thompson 2021). The Neotropical Region still holds a reasonable number of unknown *Tabanus* species, as can be seen from the number of species that have been described in recent decades (Fairchild 1983, Fairchild 1984, Gorayeb and Rafael 1984, Gorayeb and Barros 2006, Carmo and Henriques 2019). This paper aims to describe a new species of Tabanidae, *Tabanusrondoniensis* sp. n., contributing to the increase in knowledge of this family in Brazil.

## Materials and methods

The material studied comes from recent captures in the State of Rondônia. The material will be deposited in the Invertebrate Collection of the National Institute for Amazonian Research (INPA). All specimens (five females) were firstly preserved in alcohol 100% , the thorax and abdomen are somewhat crumpled and the pilosity is partly missing. Traditionally, external characters are sufficient to determine species in *Tabanus* (Fairchild 1983, Fairchild 1984, Carmo and Henriques 2019) and the use of terminalia has not been very productive below the genus level in Tabanidae (Krolow and Henriques 2009, Krolow and Henriques 2010). For these reasons, the specimens were not dissected. The morphological terminology follows Cumming and Wood (2017).

Specimens were examined and digitally photographed through a stereomicroscope LEICA M205C coupled with a LEICA DFC 295 camera and the images were processed using the software Leica Application Suite LAS V3.6. Frons indices: Frontal index = frons height/ frons width at base; Divergence index = frons width at vertex/ frons width at base.

## Taxon treatments

### Tabanus rondoniensis

Henriques, Krolow, Zamarchi & Camargo sp. n.

66894B91-994C-5945-B5B2-1ADE54A64588

B66F9CEA-F03D-45CB-80FF-67E66D55A9EF

#### Materials

- **Type status:** Holotype. **Occurrence:** recordedBy: T. Zamarchi; individualCount: 1; sex: female; **Taxon:** scientificNameID: urn:lsid:zoobank.org:act:B66F9CEA-F03D-45CB-80FF-67E66D55A9EF; order: Diptera; family: Tabanidae; genus: Tabanus; specificEpithet: *rondoniensis*; taxonRank: species; scientificNameAuthorship: Henriques, Krolow, Zamarchi and Camargo; **Location:** country: Brazil; stateProvince: Rondônia; municipality: Monte Negro; locality: LHC35-Sebastião; locationRemarks: transliteration: "BRAZIL, *Rondônia*, Monte Negro, LHC35-Sebastião, 10°09'47"S, 63°19'27"W, vii.2018, NZI trap, T. Zamarchi leg."; verbatimCoordinates: 10°09'47"S, 63°19'27"W; decimalLatitude: -10.163055555556; decimalLongitude: -63.324166666667; georeferenceProtocol: Google Earth; **Identification:** identifiedBy: AL Henriques, TK Krolow, TBO Zamarchi and LMA Camargo; **Event:** samplingProtocol: NZI trap; eventDate: 2018-07; **Record Level:** type: Holotype; institutionCode: INPA
- **Type status:** Paratype. **Occurrence:** recordedBy: T. Zamarchi; individualCount: 1; sex: female; **Taxon:** scientificNameID: urn:lsid:zoobank.org:act:B66F9CEA-F03D-45CB-80FF-67E66D55A9EF; order: Diptera; family: Tabanidae; genus: Tabanus; specificEpithet: *rondoniensis*; taxonRank: species; scientificNameAuthorship: Henriques, Krolow, Zamarchi and Camargo; **Location:** country: Brazil; stateProvince: Rondônia; municipality: Monte Negro; locality: P1-Argeu; locationRemarks: transliteration: “BRAZIL, *Rondônia*, Monte Negro, P1-Argeu, 10°28'27"S, 63°15'18"W, viii.2019, biting horse (one female)”; verbatimCoordinates: 10°28'27"S, 63°15'18"W; decimalLatitude: -10.474167; decimalLongitude: -63.255; georeferenceProtocol: Google Earth; **Identification:** identifiedBy: AL Henriques, TK Krolow, TBO Zamarchi and LMA Camargo; **Event:** samplingProtocol: biting horse; eventDate: 2019-08; **Record Level:** type: Paratype; institutionCode: INPA
- **Type status:** Paratype. **Occurrence:** recordedBy: T. Zamarchi; individualCount: 1; sex: female; **Taxon:** scientificNameID: urn:lsid:zoobank.org:act:B66F9CEA-F03D-45CB-80FF-67E66D55A9EF; order: Diptera; family: Tabanidae; genus: Tabanus; specificEpithet: *rondoniensis*; taxonRank: species; scientificNameAuthorship: Henriques, Krolow, Zamarchi and Camargo; **Location:** country: Brazil; stateProvince: Rondônia; municipality: Monte Negro; locality: P5-Necivaldo; locationRemarks: transliteration: “BRAZIL, *Rondônia*, Monte Negro, P5-Necivaldo, 10°06'1"S, 63°16'56"W, 2019-07, malaise in the forest (one female)”; verbatimCoordinates: 10°06'21"S, 63°16'56"W; decimalLatitude: -10.105833333333; decimalLongitude: -63.282222222222; georeferenceProtocol: Google Earth; **Identification:** identifiedBy: AL Henriques, TK Krolow, TBO Zamarchi and LMA Camargo; **Event:** samplingProtocol: malaise in the forest; eventDate: viii.2019; **Record Level:** type: Paratype; institutionCode: INPA
- **Type status:** Paratype. **Occurrence:** recordedBy: D. Mendes, F.F. Xavier, A. Agudelo, J.A. Rafael; individualCount: 2; sex: female; **Taxon:** scientificNameID: urn:lsid:zoobank.org:act:B66F9CEA-F03D-45CB-80FF-67E66D55A9EF; order: Diptera; family: Tabanidae; genus: Tabanus; specificEpithet: *rondoniensis*; taxonRank: species; scientificNameAuthorship: Henriques, Krolow, Zamarchi and Camargo; **Location:** country: Brazil; stateProvince: Rondônia; municipality: Porto Velho; locality: ESEC Três Irmãos; locationRemarks: transliteration: “BRAZIL, *Rondônia*, Porto Velho, ESEC Três Irmãos, 09°00'09"S, 64°32'40"W, 2017-08, malaise trap, D. Mendes, F.F. Xavier, A. Agudelo, J.A. Rafael leg. (two females)”; verbatimCoordinates: 09°00′09″S; 64°32′40″W; decimalLatitude: -09.0025; decimalLongitude: -64.544444444444; georeferenceProtocol: Google Earth; **Identification:** identifiedBy: AL Henriques, TK Krolow, TBO Zamarchi and LMA Camargo; **Event:** samplingProtocol: malaise trap; eventDate: viii.2017; **Record Level:** type: Paratypes; institutionCode: INPA

#### Description

**Holotype female**. Length: 15.2 mm. Wing: 13 mm.

**Figure 1.**
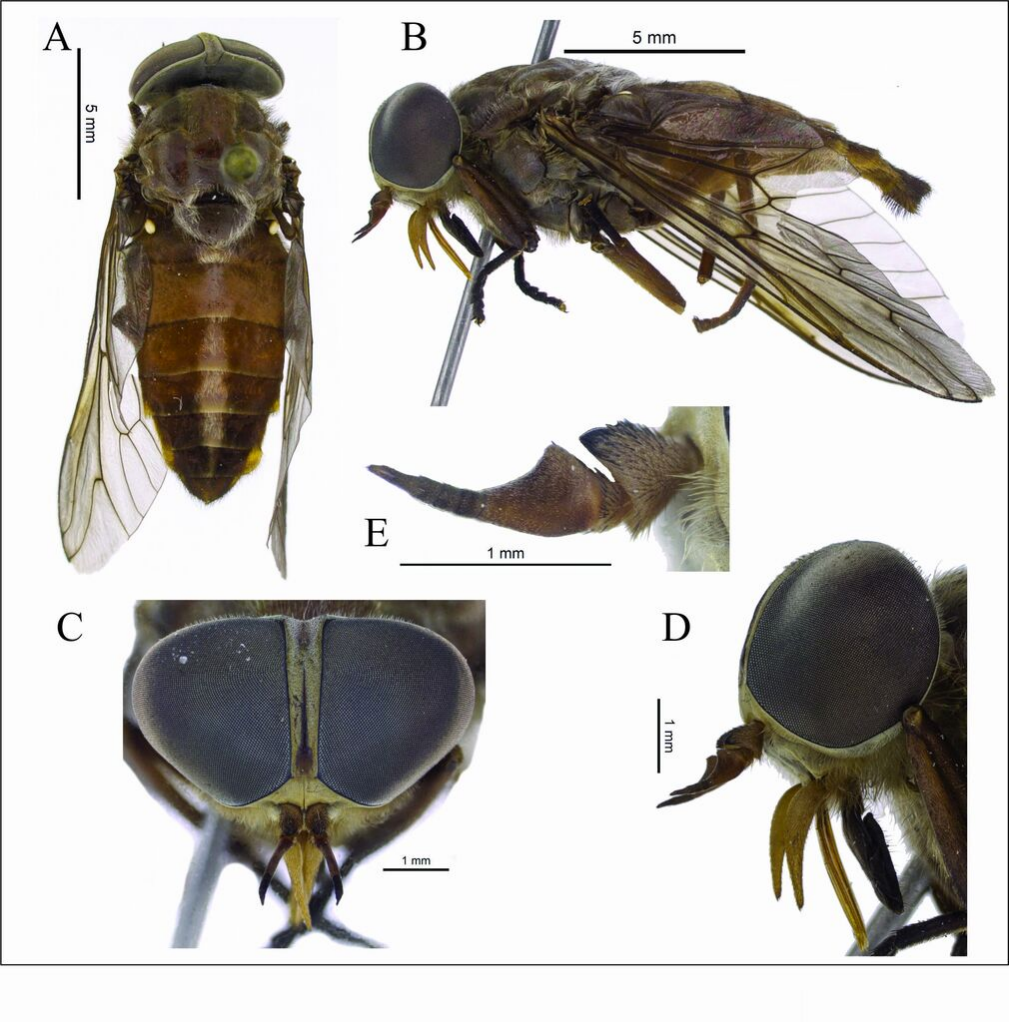
*Tabanusrondoniensis* sp. n. holotype female. **A** habitus, dorsal view; **B** habitus, lateral view; **C** head, frontal view; **D** head, lateral view; **E** left antenna.

Head. Eyes unicolorous, brown in life, glabrous. Frons moderately narrow (frontal index 5.7), somewhat divergent above (divergence index 1.5) (Fig. 1C). Frontal callus brown; ocellar triangle at vertex vestigial. Subcallus, parafacial, clypeus and gena with brownish-grey pruinescence; hairs of gena yellowish-brown. Antenna brownish, darker towards the apex (Fig. 1D-E). Scape inflated and produced dorsally, cap-like, wider than first flagellomere (basal plate). Palpus yellowish-brown, first segment with long yellowish hairs, second segment predominantly with yellowish hairs and some black. Prementum dark brown. Labellum black, membranous.

Thorax. Scutum brown with mixed yellowish and black hairs. Notopleuron concolorous with scutum, covered with black hairs, white hairs dorsally. Prescutellum black, surrounded by white hairs that are also distributed throughout the brown scutellum. Pleuron and coxae brown with grey pruinescence and pale hairs; dark hairs in the upper half of the anepisternum. Legs brown predominantly with yellowish hairs; black hairs in the apical half of fore tibia, apex of mid- and hind tibia and all tarsi. Wing with normal venation, a strong angle at fork of R_4+5_; faintly smoky with brownish shades along of the veins in the anterior half. Pterostigma weakly brown tinted.

Abdomen. Tergites brown with black hairs, except for large white median connected triangles of hairs and pruinescence on tergites 2–5, on tergite 6 a median pale line. Sides of tergites paler with yellowish hairs. Sternites brown with yellowish hairs, except sternite 7, black haired.

##### Male

Not collected (unknown).

##### Variations

Length 15 – 17 mm. Frontal index 5.5 – 6. Divergence index 1.5 – 1.8. Although many hairs are absent, variations in the colour pattern were not diagnosed.

#### Diagnosis

A brownish medium-sized species with unpatterned eyes, a well marked black pilose prescutellar spot framed and margined with white hairs (Fig. 1A-B). Antennal scape inflated, wider than first flagellomere. Abdomen with a median row of large pale pilose and pruinose connected triangles on tergites 2–5, tergite 6 reduced to a narrow stripe.

#### Etymology

The specific name refers to the State of Brazil where the specimens was recorded, Rondônia.

#### Distribution

Brazil: Rondônia.

##### Biology

Haematophagy was confirmed through the capture of a female performing a blood meal in the horse.

## Discussion

The new species described here possibly is related to *T.nebulosus* species-group, which has nine species and one subspecies, all Neotropical with occurrence records for Brazil or neighbouring countries, with the exception of *T.punctipleura* Hine, 1920, restricted to Costa Rica and Panama. According to Fairchild (1984) *T.nebulosus* species-group “has species with a moderately narrow and generally nearly parallel sided frons with slender clavate or ridge-like callus and fairly prominent black pilose prescutellar spot”. *T.rondoniensis* sp. n. can be distinguished from the other species of the *T.nebulosus* group by the diagnostic characters (unpatterned eyes, antennal scape inflated, wider than first flagellomere, a black pilose prescutellar spot, a median row of large pale pilose and pruinose connected triangles on tergites 2–5) and, additionally, by the wing without contrast marks; abdomen without dorso-lateral pale patches or dark median integumentary markings; palpus not reduced; unpainted costal cell and brown legs. *Tabanuscomosus* Stone, 1944, which has an enlarged scape and divergent frons above, can be distinguished by the presence of dorsolateral pale patches on the tergites, absent in *T.rondoniensis*. *T.rondoniensis* can be confused with *T.rubripes* Macquart, 1838, which also has a black prescutellum and light median triangles on the abdomen, but it has two-banded eyes, frons narrower (frontal index ca. 9), a long appendix on the fork of vein R_4+5_, cell r_5_ narrowed at apex or closed and the median pale triangles in the abdomen are not connected. Currently, 26 species of *Tabanus* have been registered for the State of Rondônia, of which three belong to the *T.nebulosus* species-group (Henriques and Gorayeb 1993, Henriques 1997,Coscarón and Papavero 2009, Carmo and Henriques 2019). *T.rondoniensis* is the 27th species of *Tabanus* and the fourth of the *T.nebulosus* species-group for the State of Rondônia.

## Supplementary Material

XML Treatment for Tabanus rondoniensis
